# Bright future for physiological research in *Physiological Reports*

**DOI:** 10.1002/PHY2.1

**Published:** 2013-06-27

**Authors:** Susan Wray

**Affiliations:** Department of Cellular and Molecular Physiology, Institute of Translational MedicineUniversity of Liverpool


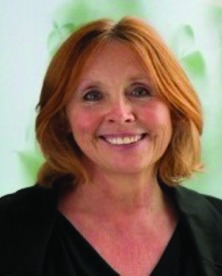


The unique collaboration of the two major physiological societies, The Physiological Society and the American Physiological Society, in their first joint publishing venture, highlights that something of major importance is happening. That event is the launch of this journal, *Physiological Reports*. This online-only journal will be the flagship open access vehicle for the two societies, adding a rigour and substance that can surpass other open access journals, and providing a venue for physiologists to publish research that constitutes an important contribution to the field.

*Physiological Reports* is international in its reach and broad in its scope. The journal will publish good, solid peer-reviewed research in basic and translational, physiology and allied disciplines, and take the lead in keeping our discipline dynamic, accelerating discovery and giving physiology a competitive edge. So, what does this mean?

No matter from where in the world you may be reading this editorial, it is likely that you are aware of the drive throughout academic publishing towards publishing models that make content accessible online to all, without delay. *Physiological Reports* brings to the physiological literature all that is best about public access. Within days of your paper being accepted, it can be read by anyone, anywhere with internet access, at no cost to the reader. This benefits you as an author: drawing attention to your work, allowing you to distribute it to your peers, and enabling the results of your research to be freely available to current and potential grant funders in a respected, peer-reviewed publication. It also benefits our discipline: *Physiological Reports* will give the physiological community speedier access to the latest research and reviews in their area. New concepts will emerge faster and can be shared by all.

Importantly, *Physiological Reports* is not limited to a narrow or trendy area of physiology; we are interested in all aspects of physiology. We are interested in cellular and molecular studies as well as intact tissues, model organisms and translational studies. It is important that in multidisciplinary studies, such as translational ones, physiologists have a natural home for their data. After all, physiology is a key underpinning discipline in translational studies. We welcome the work of biomedical scientists, whose studies incorporate physiological findings. We will also publish papers with negative data or confirmatory results, as long as they are well conceived, as these studies should benefit our community. We do not make a subjective judgement on priority and importance, simply on scientific rigour, and we provide a platform for all physiological research whose publication will be of benefit to the community.

*Physiological Reports* will bring to readers enhanced features that are possible with an online only journal. You will see that our website is slick, attractive and interactive. We plan to engage our authors and readers with social media and encourage constructive feedback on the articles we publish.

We also think you will find our submission and peer–review process friendly and we aim to give authors a decision very quickly. Our internationally distinguished and geographically-balanced board will provide feedback on your paper in a fair and constructive manner, focusing on essential enhancements to improve clarity and remove ambiguity, avoiding requests for significant re-work.

Our goal is to convince you by our processes and outcomes, our attractive website and its features, and the added value we will bring to your papers, that *Physiological Reports* is where you should be sending your research. As a further inducement, the first 100 papers to be accepted will incur no publication charges. We look forward to you joining us as readers and authors in this exciting venture and we welcome your feedback.

